# A Confirmatory Method Based on HPLC-MS/MS for the Detection and Quantification of Residue of Tetracyclines in Nonmedicated Feed

**DOI:** 10.1155/2016/1202954

**Published:** 2016-08-09

**Authors:** Rosa E. Gavilán, Carolina Nebot, Maria Veiga-Gómez, Paula Roca-Saavedra, Beatriz Vazquez Belda, Carlos M. Franco, Alberto Cepeda

**Affiliations:** Department of Analytical Chemistry, Nutrition and Bromatology, Faculty of Veterinary Medicine, University of Santiago de Compostela, 27002 Lugo, Spain

## Abstract

The Commission Regulation 574/2011/EC set up maximum levels of coccidiostats and histomonostats in nonmedicated feed as a consequence of carry-over during manufacturing. Carry-over takes place from medicated to nonmedicated feed during feed production. Similar contamination could also occur for other pharmaceuticals such as tetracyclines, a group of antibiotics commonly employed in food production animal. The objective of this work is to present a simple and fast method for the simultaneous detection of four tetracyclines (chlortetracycline, doxycycline, oxytetracycline, and tetracycline) in nontarget feed at a *μ*g/kg level. Validation of the method was performed according to the guideline included in the Commission Decision 2002/657/EC for official method. The validated method was successfully applied to 50 feed samples collected from different milk farms and 25 samples obtained from feed manufacturers. While oxytetracycline was the tetracycline most frequently detected, chlortetracycline was the analyte measured at the highest concentration 15.14 mg/Kg. From 75 nonmedicated feed analysed 15% resulted to be positive for the presence of one tetracycline.

## 1. Introduction

The demand for food of animal origin increases each year. To satisfy this demand, livestock production within the European Union (EU) in 2011 was approximately 10 million heads of goats, 80 million heads of sheep, 80 million heads of cattle, and 150 million heads of pigs [1]. To produce safe and nutritional food products, the animals need to be in good health. Like humans, animals also get sick and need medicines. Even though veterinary medicines contribute to improving and maintaining animal health, administration of these medicines by the farmer is carried out under licence via a veterinarian. The amount of drugs employed in food production estimated by Kools et al. was 6051 t, with antibiotics the most frequently used class of drug (5393 t) [2]. The groups of tetracyclines and *β*-lactams were used in high amounts and antiparasitic agents (194 t) were the second most frequently used class of drug.

Intensively produced animals are often fed with concentrated feed, a mixture of various materials (oats, wheat, barley, rye, cottonseed, and crambe) and additives. Antibiotic and antiparasitic agents are the classes of drugs most commonly administrated. Antibiotic sulphonamides, tetracyclines, and *β*-lactam are the most frequently used antibiotics, and coccidiostats and ivermectin are the most frequently used antiparasitic agents. Coccidiostats and histomonostats are a group of antiparasitic agents that have been shown to be persistent during the manufacture of feed and carry-over of this type of drugs has been demonstrated. The EU introduced maximum levels for these substances in nontarget feed in 2009 [3]; this regulation was later modified for some coccidiostats by the Regulation EC/574/2011 [4].

Cross contamination between medicated and nonmedicated feed could occur with any type of drug added to the feed, not only to coccidiostats, particularly when the cleaning process between batches is inefficient. Recently, a study conducted by Stolker et al. in the Netherlands confirmed that nonmedicated feed batches were contaminated with antibiotic residues such as tetracyclines, penicillins, and sulfonamides [5]. From 140 samples analysed, 87% tested positive for antibiotics with a concentration of 0.1–154 mg/kg. The fact that antibiotics could be present as contaminants in feed without the farmers' knowledge implies that withdrawal times will not be considered and antibiotic residues could remain in animal products (meat, eggs, milk, honey, seafood, and fish), in addition to the development of antibiotic resistant bacteria [6].

Due to the problems related to food safety, the authorities involved regularly monitor the presence of veterinary drugs in food of animal origin (eggs, milk, muscle, and liver). Controls on the water and food consumed by the animals have also been implemented, but the analysis conducted only evaluated the presence of substances such as pesticides [7, 8], nitrofurans [9], and mycotoxins [10, 11]. Methods based on HPLC-MS/MS detection are considered confirmatory methods because these types of methods provide full or complementary information, enabling substances to be unequivocally identified and if necessary quantified at the level of interest, according to the European Commission Decision 2002/657/EC [12]. Therefore, it is recommended to use confirmatory methods to detect the presence of antibiotics in nontarget feed. Few methods can be found in the scientific literature with these characteristics. Van Poucke et al. reported a method for the analysis of zinc bacitracin, spiramycin, tylosin, and virginiamycin with quantification limits below 500 *μ*g/kg [13]. Boscher et al. published a multiclass method for the analysis of 33 analytes for 14 groups of antibiotics (including tetracyclines, quinolones, penicillins, ionophore coccidiostats, macrolides, and sulphonamides) with quantification limits of 3.8–65.0 *μ*g/kg [14]. More recently, a method reported by Stolker et al. was able to measure tetracyclines, macrolides, sulphonamides, and penicillins [5].

Of the eight forms of commercially available tetracyclines, four are frequently used in food animal production (chlortetracycline, doxycycline, oxytetracycline, and tetracycline). Maximum residue limits (MRL) for these four tetracyclines and their three epimers have been introduced for various foods of animal origin, including eggs, muscle, and milk [15]. As maximum levels (ML) for these substances in feed samples have not been established, their complete absence is expected. Concentrations of tetracyclines in medicated feed are variable and depend on the target animal, with dosage rates between 25 and 700 mg/kg. Therefore, carry-over should be expected in the batch of feed manufactured after a medicated feed. Stolker et al. reported that 100% of samples from the first batch of feed produced after the manufacture of medicated feed were contaminated with tetracyclines, with concentrations of 0.5–154 mg/Kg [5]. Based on these results, carry-over contamination is also expected for the other two commonly used tetracyclines (chlortetracycline and tetracycline) as they have similar chemical properties [16, 17].

Based on the common use of tetracyclines in food animal production and the absence of confirmatory methods for the presence of the four tetracyclines in animal feed, the aim of this work is to present an HPLC-MS/MS method for the analysis of tetracyclines in nonmedicated feed samples at levels of *μ*g/kg.

## 2. Experimental

### 2.1. Chemicals, Reagents, and Stock Solutions

Disodium hydrogen phosphate dehydrate, anhydrous citric acid, ethylenediaminetetraacetic acid disodium salt (EDTA), trichloroacetic acid (TCA), and formic acid (purity > 99% by analysis) were purchased from Sigma-Aldrich (MO, USA). Tetracycline, chlortetracycline, doxycycline, and oxytetracycline (purity > 98%) and demeclocycline, used as the internal standard (IS), were supplied by Sigma-Aldrich (MO, USA).

Organic solvents, methanol and ethyl acetate, HPLC or analytical grade, were purchased from Scharlau Chemie (Barcelona, Spain) and demineralised water (resistivity 18 MU cm) was prepared in-house with a Milli-Q water system (Millipore, Bedford, MA, USA).

Mobile phase A consisted of Milli-Q water acidified to 0.04% with formic acid and mobile phase B consisted of methanol, acidified to 0.1% with formic acid. To prepare the individual stock solution of tetracycline, 20 mg of tetracycline was dissolved in 20 mL of methanol and stored at −20°C for up to six months. The intermediate solution, a mixture of tetracyclines, was prepared by diluting the stock solution of each tetracycline to a final concentration of 50 *μ*g/mL and stored at −20°C for up to one month. A standard working solution of tetracycline was prepared freshly each day by diluting the intermediate stock solution to a final concentration of 1 *μ*g/mL. For the internal standard stock solution, intermediate and working solutions were prepared and stored at −20°C for two months.

McIlvaine buffer was prepared with 10.8 g of citric acid, 10.93 g of disodium hydrogen phosphate, and 33.62 g of ethylenediaminetetraacetic acid disodium salt dihydrate (C_10_H_14_N_2_Na_2_O_8_). Each reagent was diluted first individually in approximately 100 mL of water. The EDTA solution was heated to completely dissolve the compound, avoiding reaching 50°C. Once the three reagents were completely dissolved they were mixed and the volume was made up to 1 L and the pH adjusted to pH 4.

### 2.2. Analysis by HPLC-MS/MS

HPLC-MS/MS determination of tetracyclines was performed according to a previously reported method [18]. The HPLC-MS/MS consisted of an HPLC Alliance 2795 and a MS Quattro Premier XE triple quadrupole (Waters, Manchester, UK) controlled by the software Masslynx 4.1 (Waters, Manchester, UK). The chromatographic analyses were performed by injecting 25 *μ*L of extract into a Sunfire C18 column (150 × 2.1 mm i.d., 5.0 mm) (Waters, Manchester, UK). Mobile phases A and B were mixed on a gradient mode and with a flow rate of 0.25 mL/min. Autosampler and column temperature was set at 8 and 35°C.

An electrospray ionisation (ESI) probe was set up on the triple quadrupole MS to evaporate the mobile phase coming from the HPLC and to ionise the tetracyclines. Analytes were detected in positive-ion mode and under the following conditions: capillary voltage 3 kV, source temperature 120°C, desolvation temperature 350°C, cone gas flow 49 L h^−1^, and desolvation gas flow 650 L h^−1^. As indicated in the Decision 2002/657/EC, tetracyclines were identified on the basis of their selected reaction monitoring (SRM) transition and their retention time (Rt).

### 2.3. Sample Extraction

The sample extraction procedure was based on our previous work [17]. Firstly, before sample analysis, matrix matched calibration curves were prepared. Different volumes of the tetracycline working standard solution were added to 2 g feed samples (exempt of tetracycline) and shaken in the dark for 30 min. Concentrations of tetracycline in the matrix matched feed samples were 0, 400, 800, 1200, 1600, and 1600 *μ*g/kg.

To extract the tetracyclines from the feed samples, 2 g of grounded feed, 8 mL of McIlvaine buffer, 300 *μ*L of TCA, and 0,1 mL of IS working solution were added to a 50 mL polypropylene tube. After shaking the sample in the dark for 10 min, 6 mL of ethyl acetate was added and the samples were shaken in an orbital shaker at 200 rpm for 20 min. After 15 of centrifugation at 4500 rpm, in a model 5415D centrifuge (Eppendorf, Hamburg, Germany), 2 mL of the supernatant was transferred to a 10 mL amber conical tube and evaporated to dryness, in a turboevaporator model Turbo Vap II de Zyrmark (Hopkinton, MA, USA). The final residue was dissolved in 0.5 mL of a mixture of mobile phase components (90A:10B) and vortexed. To filtrate the final extract Ultrafree-MC centrifugal filter (Millipore, MA, US) was employed and centrifuged at 9000 rpm for 10 min. The filtrate was transferred into an HPLC vial which contained a 0.3 mL microinsert and stored at −20°C; analyses were conducted within 3 days.

### 2.4. Validation according to the Decision 2002/657/EC

The guidelines used to validate the method and to interpret the results were those established in the Commission Decision 2002/657/EC. The decision establishes criteria and procedures for the validation of analytical methods to ensure the quality and comparability of analytical results generated by official laboratories.

Aspects such us trueness/recovery, precision (under repeatability and reproducibility conditions), specificity, and applicability/ruggedness/stability of the method were investigated. Trueness and the other validation parameters were assessed through recovery of additions of known amounts of tetracyclines in blank feed samples (except tetracyclines) as no certified reference material exists for this type of analysis and following the recommendation included in the Decision 2002/657/EC.

For validation one batch of matrix-matched samples was prepared with 21 samples fortified with tetracyclines at six concentrations (0, 400, 800, 1200, 1600, and 4000 *μ*g/kg). For concentrations 400, 800, and 1200 *μ*g/kg six replicated samples were prepared with only one sample for the remaining concentrations (0, 1600, and 4000 *μ*g/kg). Samples spiked with tetracyclines were shaken 10 min for homogenization. The whole procedure with 21 samples was repeated twice on two different days. Additionally, two reagent samples were prepared for control, a blank reagent (containing only the reagents) and fortified reagent (containing 1200 *μ*g/kg of tetracycline and the reagents).

To conduct the validation three batches of matrix-matched samples were prepared; each batch consisted of 21 samples fortified with tetracyclines at 0, 400, 800, 1200, 1600, and 4000 *μ*g/kg. While for levels of 400, 800, and 1200 *μ*g/kg six replicated samples were prepared, only one sample was used for levels of 0, 1600, and 4000 *μ*g/kg. After fortification, and prior to extraction, samples were shaken on an orbital shaker at 200 rpm for 10 min. After the samples extraction procedure explained above was applied. As well as the 21 matrix-matched samples, with each batch of samples two additional “samples” were prepared with reagents (no feed); one was spiked with tetracycline at 1200 *μ*g/kg (fortified reagents) and the other was not spiked with tetracyclines (blank reagent).

Additionally, 20 feed samples were analysed to determine selectivity/specificity. Ten were spiked with tetracyclines at a validation level (800 *μ*g/kg) and with 400 *μ*g/kg of three antimicrobial drugs commonly used in food animal production (sulfadiazine, sulfamethoxazol, and trimethoprim). The other ten samples were analysed without adding any veterinary drugs (Decision 2002/657/EC).

The decision limit (CC*α*) and detection capability (CC*β*) were determined as described by Freitas et al., following the Decision 2002/657/EC requirements and applying the validation level of 800 *μ*g/kg.

### 2.5. Sample Collection and Analysis

Nonmedicated feed samples were collected from 50 milk farms located in Galicia, Spain, to investigate the presence of tetracycline residues in feed that are being consumed by cows that are producing milk daily. Additionally, samples were supplied by feed manufacturers to investigate carry-over levels of tetracyclines after making medicated feed. The sampling procedures in both cases were conducted following the requirement of the Regulation 691/2013 [19]. Feed samples were stored at room temperature and in the dark with the objective of using similar store conditions compared to that in the industry and farms.

The main raw materials of the feed samples according to the labels were corn genetically modified (between 36 and 15%), soy flour produced from soya been genetically modified (present in some samples, between 4 and 38%), colza flour (present in some samples, between 3 and 47%), and barley (present in some samples, between 6 and 18%); each feed sample had a particular composition which normally depends on the manufacture.

On the other proximate composition the feed samples were crude protein (between 18 and 26%), crude fibre (between 4 and 10%), oil and fat crude (between 2.5 and 6%), crude ash (between 6 and 10%), and sodium (between 0.7 and 4%).

## 3. Results and Discussion

### 3.1. Method Optimisation

Tetracyclines have more than three hydroxyl groups that are easily ionisable by ESI to enhance their detection. Therefore, ESI is commonly used for the ionisation of tetracyclines when analysis is conducted by HPLC-MS/MS, independently of the matrix type [14, 20–24]. To optimise the MS parameters for a high MS signal response, standard solutions of 1 *μ*g/mL of individual tetracyclines were infused directly into the MS. During this procedure, one precursor ion and two product ions were selected for each tetracycline to conduct SRM analysis (Table 1). With the HPLC-MS/MS operating on SRM mode, two transitions and the retention time were used to achieve four identification points for each of the analytes, as required in the Decision 2002/657/EC. It should be highlighted that with other detection methods, such as diode array or a fluorescence detector, only one identification point is achieved [14] and more steps are required. The use of precursor and product ions identified in this research for tetracyclines was also reported by Boscher et al. 2010 for the analysis of these analytes in feed, royal jelly, and muscle [14, 20]. The separation gradient could be run at ambient temperature; however, it was observed that an increase in mobile pressure can cause the system to collapse; therefore a temperature of 35°C is recommended.

### 3.2. Optimisation of the Extraction Protocol

Based on previously reported methods for tetracycline analysis in food and feed matrices [14, 25–27] different extraction protocols were tested. Firstly, simple extractions with ethyl acetate, hexane, acetonitrile, methanol, and dichloromethane were tested. However, recoveries were low, due to the tendency of tetracyclines to form chelation complexes with different cations. Therefore, initial extraction with McIlvaine/EDTA buffer was employed and gave satisfactory results, as in previously reported methods [7, 21, 28, 29]. Tetracyclines dissolved in the McIlvaine/EDTA solution were then extracted with ethyl acetate, as these two solutions are immiscible, and separation could be easily conducted as the organic phase stays on the top layer. Other authors employ SPE cartridges such as OASIS [5] for dispersive SPE [14] instead of ethyl acetate in order to purify the extract. The use of SPE was avoided to reduce time and cost of the analysis as satisfactory results were achieved with the presented method.

Animal feed is derived from a multitude of raw materials from plant and animal origin, as well as pharmaceutical and industrial sources. As feed ingredients vary depending upon the animal, that is, poultry, swine, and cattle [30], analysis of tetracyclines in different feed types could be more complicated, particularly as the fat content will vary. The method used in this paper has been tested in feed for cattle, laying birds and chickens, rabbits, and dairy cows, and satisfactory results were obtained in all cases. These matrices were tested by preparing matrix-matched calibration curves with each type of feed, depending on the animal that was going to consume the feed.

### 3.3. Method Validation

Calibration curves to quantify concentrations of tetracyclines were obtained by spiking feed samples with the analytes at different concentrations. If linear regression coefficients (*R* ^2^) were below 0.98 the extraction procedure was repeated.

**Figure 1:**
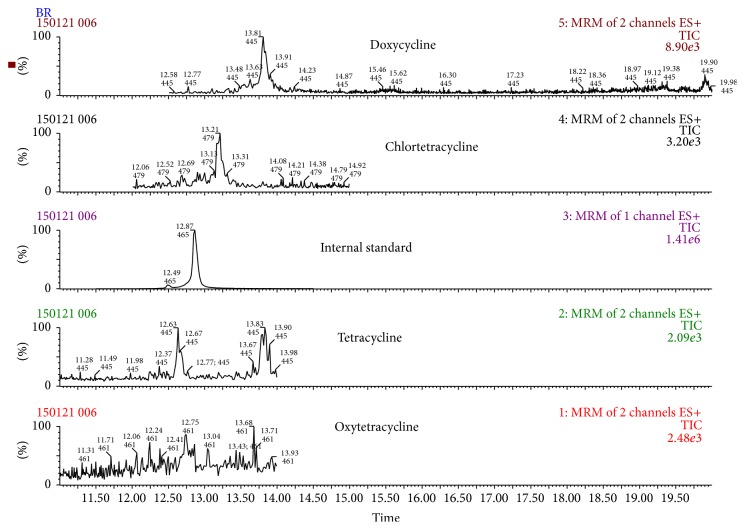
SRM chromatograms of tetracyclines in a blank sample.

**Figure 2:**
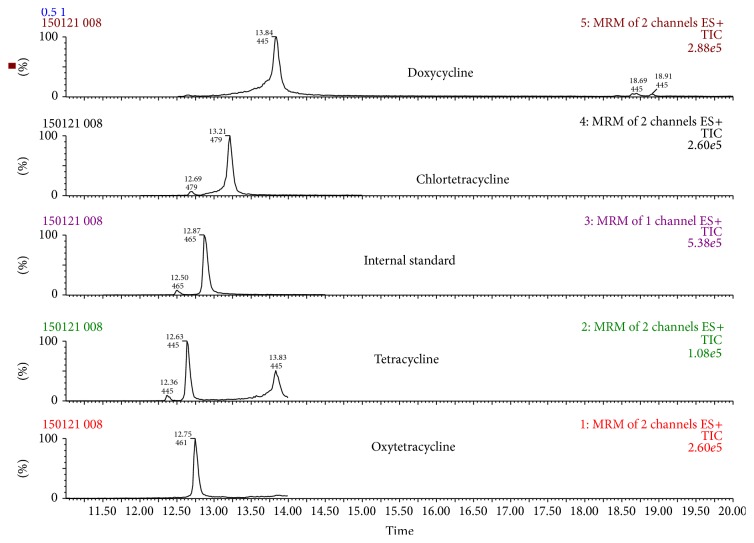
SRM chromatograms of tetracyclines in a sample fortified at 400 *μ*g/kg.

**Figure 3:**
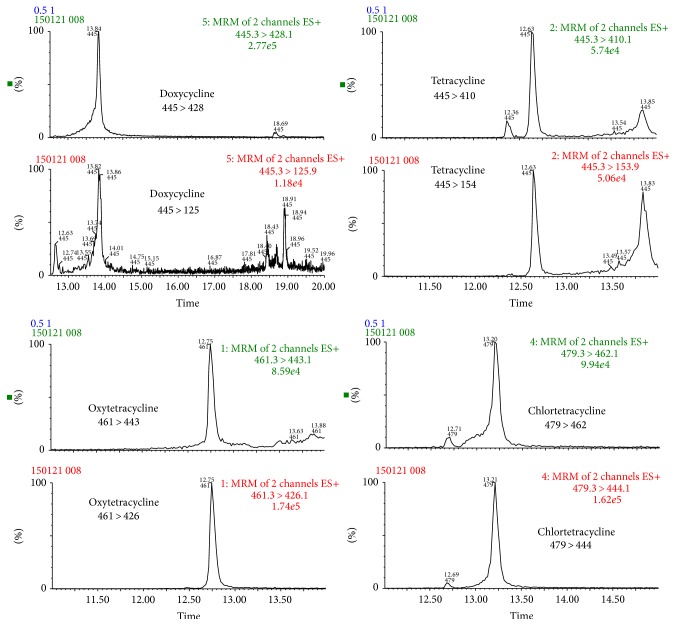
SRM chromatograms of individual tetracyclines in a sample fortified at 400 *μ*g/kg.

Even if a signal to noise ratio (S/N) higher than 10 was achieved at 300 *μ*g/kg, validation was conducted at 800 *μ*g/kg to provide acceptable results at 0.5, 1, and 1.5 times the validation level (800 *μ*g/kg) recommended by the Decision 2002/657/EC.  Figure 1 shows chromatograms of a blank sample, Figure 2 shows a blank sample spiked with all the tetracyclines at 400 *μ*g/kg, and Figure 3 shows the SRM transition employed for each tetracycline in one of the samples spiked at 400 *μ*g/kg.

Reference materials were not available; therefore trueness of the method was calculated in terms of recoveries. Results obtained during the validation are summarised in Table 2. Recovery was low compared with other reported methods. However, the advantage of the presented extraction protocol is that it does not require solid phase extraction and the four main tetracyclines can be identified and quantified simultaneously.

Results for repeatability, calculated as the mean RDS of the RSD (*n* = 6) for each concentration on each day of the validation, were below 17% for chlortetracycline, doxycycline, oxytetracycline, and tetracycline (Table 2). Results for reproducibility, calculated as the RDS of 21 samples at the same concentration, were below 23% for all tetracyclines (Table 2).

To determine selectivity/specificity, 10 blank samples and the same samples spiked with the four tetracyclines at 800 *μ*g/kg were analysed. The successful quantification of tetracyclines and the absence of interfering peaks at the retention times of each analyte demonstrated the selectivity/specificity of the method.

The limit of detection (LOD) and limit of quantification (LOQ) of the method were calculated and verified with feed samples spiked with the tetracyclines at different concentrations. Based on S/N above 3 for LOD and above 10 for LOQ in matrix-matched samples, the LOD and LOQ of the method were set at 35 and 47 *μ*g/kg for chlortetracycline, 40 and 60 *μ*g/kg for oxytetracycline, 24 and 40 *μ*g/kg for tetracycline, and 100 and 150 *μ*g/kg for doxycycline.

CC*α* and CC*β* were determined using the conditions for substances for which no permitted limit has been established. CC*α* and CC*β* were higher than LOD and LOQ for all the compounds, meaning that tetracyclines detected at a higher level than the CC*β* will be positive and levels of tetracyclines were quantifiable, without doubt. CC*α* and CC*β* for chlortetracycline, doxycycline, oxytetracycline, and tetracycline were below 400 *μ*g/kg (Table 2).

Validation results already published have shown, in some cases, higher repeatability, reproducibility, and lower LOQ, such as the work conducted by Boscher et al. who reported RSD lower than 12% and LOQ of 20 *μ*g/kg. Similarly, the method reported by Stolker et al. achieved a LOQ of 0.1 *μ*g/kg for doxycycline and oxytetracycline. However, it should be highlighted that none of the methods reported, based on the authors knowledge, have been validated according to the Decision 2002/657/EC.

### 3.4. Real Sample Collection and Analysis

From 75 feed samples investigated oxytetracycline was the tetracycline more frequently detected present in 8% of the samples (*n* = 6); its concentration range was between 90 and 400 mg/kg. On the other hand, tetracycline and doxycycline were least detected. Each tetracycline was detected in individual samples and their concentrations were 150 and 110 mg/kg. Chlortetracycline was the pharmaceutical detected at the highest concentration in this study, 15.14 mg/kg in feed samples for calves. It is important to highlight that the consumption of contaminated feed at level such as 15.14 mg/kg could cause food safety problems giving positive food samples.

## 4. Conclusion

Carry-over during feed manufacture has been proved for veterinary drugs such as coccidiostats and a similar case can be considered for tetracyclines, a group of antimicrobial agents commonly used in food animal production, due to its low cost.

The research work presents a simple and fast method for the analysis of the four tetracyclines regulated in the production of food of animal origin (chlortetracycline, doxycycline, oxytetracycline, and tetracycline). The method was validated according to the European guideline and successfully applied to 75 nonmedicated feed samples. Results showed the presence of tetracyclines in 15% of the samples, indicating that cross contamination occurs and maximum levels for tetracyclines may be required in the future.

## Figures and Tables

**Table 1:** Retention time (Rt), cone voltage (CV), collision energy, precursor, and product ions employed for ion identification.

Tetracycline	Rt (min)	Precursor > production	CV	Collision energy
Tetracycline	12.63	445 > 410	30	29
Tetracycline		445 > 154	30	27

Doxycycline	13.84	445 > 428	30	20
Doxycycline		445 > 125	30	27

Chlortetracycline	11.84	479 > 462	30	23
Chlortetracycline		479 > 444	30	23

Oxytetracycline	12.61	461 > 443	30	20
Oxytetracycline		461 > 426	30	20

Demeclocycline	12.00	465 > 448	30	17

**Table 2:** Recoveries (%), repeatability (CV%), within-laboratory reproducibility (CV%), CC*α*, CC*β*, LOD, and LOQ of tetracyclines.

Tetracycline	CC*α* (*μ*g/kg)	CC*β* (*μ*g/kg)	LOD (*μ*g/kg)	LOQ (*μ*g/kg)	Level (*μ*g/kg)	Accuracy	Repeatability	Reproducibility
Chlortetracycline	146	249	35	47	400	89	11	13
					800	91	12	13
					1200	111	17	23

Doxycycline	205	344	100	150	400	93	16	22
					800	113	15	17
					1200	109	17	19

Oxytetracycline	198	315	40	60	400	103	16	20
					800	90	12	22
					1200	103	15	20

Tetracycline	92	164	24	40	400	78	12	13
					800	95	12	13
					1200	100	10	10
